# Documentation of suicidality in connection with specialised healthcare for physical conditions: a retrospective review of somatic medical records prior to suicide in Sweden

**DOI:** 10.1136/bmjopen-2024-086633

**Published:** 2025-05-15

**Authors:** Tabita Sellin, Margda Waern, Erik Bergqvist, Nina Palmqvist Öberg, Sara Lindström, Elin Fröding, Anna Ehnvall, Åsa Westrin

**Affiliations:** 1 Faculty of Medicine and Health, University Health Care Research Center, Örebro University Hospital, Örebro University, Orebro, Sweden; 2 Department of Psychiatry and Neurochemistry, Institute of Neuroscience and Physiology, University of Gothenburg. Psychosis Department, Sahlgrenska University Hospital, Region Västra Götaland, SE-431 30 Mölndal, Göteborg, Sweden; 3 Department of Clinical Sciences, Psychiatry, Lund University. Psychiatric In-patient Clinic, Hallands Sjukhus Varberg, Region Halland, SE-432 81 Varberg, Lund, Sweden; 4 Department of Clinical Sciences, Psychiatry, Lund University. Psychitaric Clinic, Region Skåne, SE-221 85, Lund, Sweden; 5 Department of Clinical Sciences, Lund University. The Region Skåne Committee on Psychiatry, Habilitation and Technical Aids, SE-221 85, Lund, Sweden; 6 Region Jönköpings län, Jönkoping, Sweden; 7 Department of Psychiatry and Neurochemistry, Institute of Neuroscience and Physiology, University of Gothenburg. Psychiatric Outpatient Clinic, Region Halland, SE-432 43 Varberg, Göteborg, Sweden; 8 Department of Clinical Sciences, Psychiatry, Lund University. The Region Skåne Committee on Psychiatry, Habilitation and Technical Aids, SE-221 85, Lund, Sweden

**Keywords:** Suicide & self-harm, Risk Factors, Chronic Disease

## Abstract

**Abstract:**

**Objectives:**

To evaluate whether suicidality was documented prior to suicide in patients in contact with specialised somatic healthcare providers for physical conditions and to identify factors related to such documentation.

**Design and settings:**

Retrospective cohort study in which medical records from specialised somatic (non-psychiatric) healthcare services (internal medicine, infectious disease, surgery, urology, etc) in 20 of Sweden’s 21 regions were reviewed up to 2 years before suicide.

**Participants:**

Those who died by suicide in Sweden 2015 and had received specialised somatic healthcare for a diagnosed physical condition were included, n=468 (331 men and 137 women).

**The outcome variable:**

*Documentation of suicidality* (ie, death wishes, suicidal thoughts, plans, attempts and notations of known suicidality or elevated suicide risk). Potential associations of patients’ characteristics and clinical factors with the outcome were tested in logistic regression models.

**Results:**

Of the 468 patients, 111 (24%) were positive for the outcome variable *Documentation of suicidality,* regardless of whether they were assessed as suicidal or not. Elevated suicide risk was noted in 27 patients (6% of the total cohort). Multivariate logistic regression analysis showed that experience of distress (OR: 4.81; 95% CI: 1.96 to 11.81), contact with psychiatric services (OR: 4.68; 95% CI: 2.60 to 8.43), psychiatric comorbidity (OR: 4.33; 95% CI: 2.41 to 7.76) and female sex (OR: 2.91; 95% CI: 1.68 to 5.06) were independently associated with documentation of suicidality. A third (36%) had a doctor consultation in specialised somatic healthcare during their last month of life. Of these, 17% were assessed for suicidality, and elevated suicide risk was noted in 7%.

**Conclusions:**

Documentation of suicidality was observed in one quarter of patients who received specialised somatic healthcare for physical conditions and subsequently died by suicide. These results indicate a need to increase clinician awareness of suicidal issues and assessments and to integrate questions about mental health into specialised somatic practice.

**STRENGTHS AND LIMITATIONS OF THIS STUDY**

- Documentation of suicidality prior to suicide in patients in contact with specialised somatic care is little studied.
- The comprehensive medical record review provided rich data.
- There was no systematic testing of inter-rater reliability of data ratings in this study.
- Persons with physical conditions who died due to self-harm of undetermined intent were not included, which limits the generalisability to patients with at least one diagnosed physical condition and a cause of death as certain suicide.

## Introduction

 Despite the well-known connection between physical illness and suicidality,^1^,^5^ we know relatively little about the degree to which clinicians recognise and document suicidality prior to suicide. Several physical conditions are especially associated with elevated risks of suicide attempts and death by suicide, including cancers, respiratory diseases, cardiovascular diseases, multiple sclerosis, diabetes, arthritis and osteoporosis.^6^,^12^

Previous studies in psychiatric settings have found that between one-quarter and three-quarters of individuals who died by suicide had documentation of prior suicidal thoughts or behaviours.^13 14^ However, a low proportion of individuals with physical health conditions have contact with psychiatric services prior to suicide, especially older adults.^15^ Therefore, there is a need to learn more about the documentation of suicidality in care settings used by persons with physical issues in specialised somatic care before death by suicide.

During admission to a Brazilian general medicine clinic, 7% of a sample of over a thousand patients with various physical illnesses and mental conditions screened positive for the suicide ideation item (item 9) on the Patient Health Questionnaire-9 (question about passive thoughts of death or self-injury) (PHQ-9).^16^ The authors of a meta-analysis on the documentation of suicidal ideation in psychiatric and non-psychiatric populations concluded that information about suicidal ideation was often missing. However, those who did have a notation of suicidal ideation had a fourfold risk of dying by suicide during the first follow-up year compared with those not expressing suicidal ideation.^17^

The present study, with somatic medical record review of suicidality, can contribute to reducing this knowledge gap of documentation of suicidality among physically ill patients. The objectives of this study were to evaluate whether suicidality was documented prior to suicide in patients in contact with specialised somatic healthcare providers for physical conditions. This study also aimed to investigate possible associations of patients’ characteristics and clinical factors with the probability of documented suicidality.

## Methods

The present study is part of the nationwide research project entitled *Retrospective investigation of health care utilization of individuals who died by suicide in Sweden 2015*. The Swedish National Board of Health and Welfare reported 1186 certain suicides in 2015, corresponding to a rate of 11.97/100 000 inhabitants.^18^ Causes of death were registered using the International Statistical Classification of Diseases and Related Health Problems, 10th Revision (ICD-10) according to guidelines of the WHO,^19^ in which death due to intentional self-harm is coded X60–X84 and designated certain suicide. The catchment area comprised 20 of the 21 regions in Sweden with a mixture of urban and rural regions, covering a population of approximately 7.6 million inhabitants in 2015.^20^ Stockholm data was not completed and therefore unavailable at the time of this study. There were 948 certain suicides in the catchment area in 2015, which corresponds to 80% of all certain suicides in Sweden. The national project used a 24-month retrospective design to review data from the medical records of individuals who died by suicide. Data on date of death, cause of death and suicide method were obtained from the Swedish Cause of Death Register. Details on the origins of this register, as well as its composition, strengths and weaknesses, have been described.^21^ Through a structured protocol developed for this project, local healthcare teams in each county used personal identification numbers to access and review all medical records, in a uniform manner. We describe the complete methodology and data collection process in the nationwide research project in detail in our previous publications.^15 22 23^

### Study design and setting

This study, involving a cohort of patients who died by suicide, employed a retrospective design to evaluate somatic medical records. Data were derived from services provided by specialists in internal medicine; surgery and urology; ophthalmology; orthopaedic surgery; ear, nose and throat; gynaecology/obstetrics; dermatology; infectious diseases; paediatric/adolescent medicine; pain specialists; other specialist clinics and non-psychiatric emergency care. The eligibility criteria for this study were deceased by certain suicide, care contact with specialised somatic healthcare sometime in the 24 months prior to suicide, with at least one diagnosed physical condition.

### Data from somatic medical records

Data were collected from the somatic medical records of patients in specialist healthcare services within 24 months prior to suicide. The information came from emergency room presentations, hospital admissions and outpatient consultations with healthcare professionals within specialised somatic healthcare.

### Dependent variable

The dependent outcome variable *Documentation of suicidality* included any documentation in somatic medical records from both outpatient consultations and hospital admissions during the 24 months prior to suicide. Documentation came from healthcare contacts with staff of various clinical professions including doctors, nurses, psychologists, social workers, occupational therapists and more. We extracted information concerning expressions of death wishes, suicidal thoughts, plans for suicide, notes of known suicidality in somatic medical records or suicide attempts registered with ICD-10 codes X60–X84 in somatic medical records. Documentation of suicide risk as elevated was extracted from the most recent risk assessment by a doctor, which could be at any time point during the 24 months prior to suicide. All the above-mentioned data with various types of suicidality were combined to constitute the dependent outcome variable *Documentation of suicidality* (yes or no).

### Independent variables and covariates

#### Diagnoses of physical conditions

Data on registered diagnoses of physical conditions during specialist somatic care contact were collected from somatic medical records and were categorised by ICD-10 chapters I–IV and VI–XIV (code ranges A00–E90 and G00–N99). Due to the small numbers in chapters VII and VIII, these were grouped as one category (code ranges H00–H95), before analyses. For this study, a patient diagnosed with physical conditions in two or more of these categorised ICD-10 chapters was considered to have multiple physical conditions.

#### Psychiatric comorbidities

Psychiatric comorbidities were defined as having a mental or behavioural disorder according to ICD-10 chapter V (code ranges F00–F99) documented in somatic medical records, in addition to a physical condition categorised by ICD-10 chapters as described above.

#### Experience of distress

Information on the experience of distress was collected by reviewing text notes in somatic medical record data from the last contact with a doctor. A patient was considered to have experience of distress if they revealed current problems such as a crisis or difficulties in managing one’s situation. The variable was further categorised into reasons underlying these difficulties (ie, distress related to the physical condition, relationship problems, trauma (both emotional and physical), social or financial problems and overwhelming concerns about their life situation).

#### Care contacts

Data on care contacts were obtained from notes in patients’ somatic medical records, including notation of contact with psychiatric and substance use services. The date of last contact with a doctor in specialised somatic healthcare before suicide was also collected. Patients’ primary healthcare records were evaluated to determine whether the patient also had contact within primary care.

#### Sociodemographic factors

Sociodemographic data (sex, age at death, civil status, cohabitation and employment status: currently employed or retired) were collected from patients’ somatic medical records or other medical records containing sociodemographic information. Age was used as a continuous variable. All other sociodemographic variables were dichotomised.

### Statistical analysis

χ^2^ tests and independent t-tests were used to calculate the distribution of sociodemographic and clinical variables and comparisons between sexes. Mann-Whitney U test was applied (due to non-normally distributed data) to compare the number of days (median, minimum-maximum (min-max) of days) from the last contact with a doctor in somatic specialist care to suicide and comparisons between sexes.

Associations of sociodemographic factors and clinical variables with the outcome variable *Documentation of suicidality* (yes=1, no=0) within 24 months before suicide were determined by calculating unadjusted and adjusted OR and 95% CIs using binomial logistic regression models (standard method). In the univariate logistic regression analyses, ORs and CIs were estimated for sociodemographic variables (ie, sex, age, civil status, currently employed) and clinical variables (ie, experience of distress, care contacts with psychiatric services, care contact with substance use services, psychiatric comorbidity, diagnoses of a physical condition and multiple physical conditions according to ICD-10 chapters). All significant variables (p<0.05) in univariate analyses were included in the multivariable analysis, with all independent variables or covariates entered into one model simultaneously. All variables used in logistic regression models fulfilled the required assumptions for these analyses. The statistical analyses were performed using SPSS statistical software, V. 27 (IBM, New York, USA), with p<0.05 considered statistically significant.

### Patient and public involvement

None.

## Results

### Participants

Of the 948 individuals in the catchment area who died by certain suicide from 1 January to 31 December 2015, 630 (66.5%) had been in contact with specialised somatic healthcare at some point during the last 24 months of life. Patients who died by suicide whose only specialised somatic healthcare for physical conditions was in connection with suicide attempts or injuries of undetermined intent (n=77), and patients with diffuse symptoms not diagnosed as a physical condition (n=85) did not fulfil inclusion criteria and were excluded from this study. In all, 468 persons, corresponding to 49.4% of all certain suicides in the catchment area, were included in the present study (331 men and 137 women).

Sociodemographic characteristics and care contacts in addition to specialised somatic healthcare, as well as comparisons between sexes, are summarised in table 1. Age at death ranged from 13 to 95. Women were on average 6 years younger than men, and a greater proportion of women had care contacts with psychiatric services than men. About a third lived with a spouse or partner, about a quarter were employed and more than a third were retired at the time of death. Significantly more men than women were retired.

**Table 1:** Sociodemographic characteristics and healthcare contacts beside specialist somatic healthcare of all subjects (n=468)

	All	Men	Women	P value*	Test statistics
Sociodemographic characteristics†:					
Men/women, n (%)	468	331 (70.7)	137 (29.3)	-	–
Age at suicide, m (SD)	58.3 (17.9)	60.1 (17.2)	53.9 (18.8)	0.001	*t=3.48*
Civil status cohabitation, yes, n (%)‡	161 (34.4)	121 (36.6)	40 (29.2)	not significant	
Currently employed, yes, n (%)§	88 (18.8)	55 (16.6)	33 (24.9)	not significant	
Retired (age ≥65), yes, n (%)¶	180 (38.5)	140 (42.3)	40 (29.2)	0.01	χ^2^=7.02
Other healthcare contacts:					
Psychiatric services, yes, n (%)**	130 (27.8)	76 (23.0)	54 (39.4)	0.001	χ^2^=13.08
Substance use services, yes, n (%)**	25 (5.3)	15 (4.5)	10 (7.3)	not significant	
Primary care, yes, n (%)††	426 (91.0)	301 (90.9)	125 (91.2)	not significant	

* P values comparing mean age at suicide in men and women determined using independent samples t-test; *p* values comparing categorical variables (eg, civil status, currently employed, contacts in healthcare settings) determined by χ2 tests, with one degree of freedom (df=1).

† Sample size (n), mean (m), SD.

‡ Civil status cohabitation yes: living with a spouse/partner. Information about civil status missing for 96 patients.

§ Currently employed yes: having part-time or full-time employment. Information about employment missing for 124 patients.

¶ Four of the retired were working part-time. Information about retirement missing in 37 patients in age ≥65 was recoded as retired.

** From review of somatic medical records of known care contact with other healthcare services.

†† From review of primary care records.

More than half of the patients (54.9%) had contacts with internal medicine services and 40.6% with surgery and urology services. Further, 29.7% had contacts with emergency services, 22.6% with ophthalmology services, 22.4% with orthopaedic services, 15.4% with ear, nose and throat services, 7.3% with gynaecology/obstetrics services, 7.3% with dermatology services, 6.4% with infectious disease services, 1.3% with pain services, 0.4% with paediatric/adolescent services and 17.9% with other specialist clinics. Of the 468 patients, 21 (4.5%) had documented contact with somatic emergency care only. These patients with emergency care only differed from the other patients within specialised somatic clinics in only one of the examined sociodemographic factors; they had significantly higher employment rates than the remaining 447 patients (56.3% vs 24.1%, χ^2^=*8.29, p<0.01*).

Suicide occurred a median time of 64 days (min-max 0–730 days) of the last contact with a doctor in a specialised somatic healthcare setting. This figure was similar in men (63.5 days) and women (66 days). Patients with documentation of suicidality had significantly lower median time from the last consultation with a doctor in specialised somatic care to suicide (24 days, min-max 0–577) in comparison to the group without such documentation (86 days, min-max 0–730), Mann-Whitney U test: p<*0.001*).

### Physical conditions

The most common physical conditions recorded in the somatic medical records were diseases of the circulatory, musculoskeletal and digestive systems. Neoplasms were noted in only 16% (table 2).

**Table 2:** Physical conditions by ICD-10 chapters recorded in somatic medical records prior to suicide (n=468)

ICD-10 chapters with codes*	n (%)
I. Certain infectious and parasitic diseases (codes A00–B99)	45 (9.6)
II. Neoplasms (codes C00–D48)	75 (16.0)
III. Diseases of the blood and blood-forming organs and /…/ the Immune mechanism (D50–D89)	17 (3.6)
IV. Endocrine, nutritional and metabolic diseases (codes E00–E90)	90 (19.2)
VI. Diseases of the nervous system (codes G00–G99)	75 (16.0)
VII/VIII. Diseases of the eye and adnexa/ear and mastoid process (codes H00–H95)	107 (22.9)
IX. Diseases of the circulatory system (codes I00–I99)	148 (31.6)
X. Diseases of the respiratory system (codes J00–J99)	62 (13.25)
XI. Diseases of the digestive system (codes K00–K93)	110 (23.5)
XII. Diseases of the skin and subcutaneous tissue (codes L00–L99)	41 (8.8)
XIII. Diseases of the musculoskeletal system and connective tissue (codes M00–M99)	123 (26.3)
XIV. Diseases of the genitourinary system (codes N00–N99)	78 (16.7)

Diseases are presented as the number (n) and per cent (%) of patients with the physical conditions, as determined by the specified chapters in the International Statistical Classification of Diseases, 10th Revision (ICD-10).

* Individual patients may have physical conditions in multiple ICD-10 chapters.

ICD-10, International Statistical Classification of Diseases and Related Health Problems, 10th Revision.

Gender comparisons revealed that the proportion of diseases of the circulatory system was higher in men compared with women (34.7% vs 24.1%, χ^2^=*5.09, df=1, p*=*0.05*), while the proportion with diseases of the genitourinary system was significantly lower in men (13.6% vs 24.1%, χ^2^=*7.68, df=1, p=0.01)*. Of the 468 patients, 206 (44.0%) had been diagnosed with one of the physical diseases in ICD-10 chapters I–IV and VI–XIV, while 129 (27.6%) had been diagnosed with diseases in two ICD-10 chapters and 133 (28.4%) patients had been diagnosed with diseases in three or more (non-psychiatric) ICD-10 chapters.

### Psychiatric comorbidities

Psychiatric comorbidities were documented with ICD-10 chapter V (F diagnosis) in somatic medical records for slightly over one-third of women and one-fourth of men registered in this study, but these proportions did not differ significantly between women and men (36.5% vs 27.8%, χ^2^*=3.47, df=1, p=0.062)*.

### Experiences of distress

Experience of distress was retrieved from text notes in the somatic medical records for the 2-year period preceding suicide in 34 (7.3%) patients. The proportions of patients with mention of distress did not differ between women and men (10.2% vs 6.0%, χ^2^=*2.51*, *df=1, p=0.11*). Moreover, the mean ages of patients with and without documented distress did not differ significantly (60.7 vs 58.1 years, *t=0.82, p=0.82*). An indication of distress was not related to any specific physical condition, nor care contact with any specific somatic care services (data not shown). Of the 34 patients with indications of distress, 13 (38%) experienced distress related to their physical condition, including information about a diagnosis, worsening of symptoms or severity of symptoms. Nine (26%) patients had relationship problems, five (15%) experienced distress due to trauma (both emotional and physical), five (15%) had social or financial problems and two (6%) had overwhelming concerns about their life situation.

### Documentation of suicidality

Of the 468 patients, 111 (23.7 %) had documentation of suicidality. The distributions of the various types of suicidality that occurred at any point in time during the final 24 months of life showed significant sex differences in all types of suicidality, except for suicide plans. Overall, women had a significantly higher rate of documentation of suicidality compared with men (40.1% vs 16.9%) (table 3). The somatic medical records also showed that 168 (35.9%) of the 468 patients had care contact with a doctor during their last month of life in specialised somatic healthcare. In 29 (17.3%) of the 168 patients, the doctor recorded an assessment of mental status and suicide risk. Elevated suicide risk was noted for 11 of them (7% of those with consultation).

**Table 3:** Suicidality noted in the somatic medical records of all who died by suicide in 2015

	Alln (%)	Menn (%)	Womenn (%)	χ^2^*	P value*
Death wishes	34 (7.3)	16 (4.8)	18 (13.1)	9.92	0.002
Suicide-related thoughts	40 (8.5)	21 (6.3)	19 (13.9)	7.02	0.008
Suicide plans	21 (4.5)	11 (3.3)	10 (7.3)	3.57	0.059
Suicide attempts†	42 (9.0)	21 (6.3)	21 (15.3)	9.57	0.002
Notes of elevated suicide risk	27 (5.8)	13 (3.9)	14 (10.2)	7.06	0.008
Notes of known suicidality	82 (17.5)	38 (11.5)	44 (32.1)	28.55	<0.001
Any documentation of suicidality	111 (23.7)	56 (16.9)	55 (40.1)	28.89	<0.001

* P values comparing occurrence of suicidality between men and women determined by χ2 tests, with one degree of freedom (df=1).

† Suicide attempts documented as ICD-10 X60–X84 (intentional self-harm) in somatic medical records during the last 2 years of life.

### Factors associated with documentation of suicidality

Univariate analyses showed that two sociodemographic factors (ie, female sex, younger age at death) and six clinical factors (ie, experience of distress, contact with psychiatric services, contact with substance use services and specified diseases in ICD-10 chapter I (codes A00–B99): infectious/parasitic diseases, chapter X (codes J00–J99): Diseases of the respiratory system, and chapter V (codes F00–F99): psychiatric comorbidity, were significantly associated with documented suicidality during the last 24 months of life (table 4). Multivariate analysis showed that female sex, experiences of distress, contact with psychiatric services and psychiatric comorbidity were independently associated with documented suicidality.

**Table 4:** Univariate and multivariate logistic regression analyses of sociodemographic and clinical factors associated with documented suicidality

	Total samplen=468	Documentation of suicidality: yes, n=111(% within subgroup)	UnivariateOR (95% CI)	P value	MultivariateOR (95% CI)	P value
Sex, n (%)						
Men	331 (70.7)	56 (50.5)				**<0.001**
Women	137 (29.3)	55 (49.5)	**3.29(2.11to5.15)**	**<0.001**	**2.91(1.68to5.06)**	
Age at death, years (SD)	58.3 (17.9)	53.5 (18.6)	**0.98(0.97to0.99)**	**0.001**	0.99 (0.98 to 1.01)	0.476
Civil status*, n (%)						
Cohabiting	161 (34.4)	34 (30.6)	0.72 (0.45 to 1.18)	0.191		
Currently employed*, n (%)	88 (18.8)	16 (14.4)	0.55 (0.30 to 1.00)	0.050		
Experience of distress, n (%)†	34 (7.3)	19 (17.1)	**4.71(2.30to9.63)**	**<0.001**	**4.81(1.96to11.81)**	**<0.001**
Care contacts with psychiatric services, n (%)‡	130 (27.8)	71 (64.0)	**8.97(5.56to14.45)**	**<0.001**	**4.68(2.60to8.43)**	**<0.001**
Care contacts with substance use services, n (%)‡	25 (5.3)	16 (14.4)	**6.51(2.79to15.20)**	**<0.001**	1.84 (0.68 to 4.99)	0.228
Mental or behavioural disorder (ICD-10 chapter V, n (%)§	142 (30.3)	76 (68.5)	**9.57(5.92to15.49)**	**<0.001**	**4.33(2.41to7.76)**	**<0.001**
Certain infectious and parasitic diseases (ICD-10 chapter I, n (%)	45 (9.6)	19 (17.1)	**2.63(1.39to4.96)**	**<0.001**	1.39 (0.62 to 3.10)	0.428
Neoplasms (ICD-10 chapter II, n (%)	75 (16.0)	14 (12.6)	0.70 (0.38 to 1.31)	0.264		
Diseases of blood/immune mechanism (ICD-10 chapter III, n (%)	17 (3.6)	6 (5.4)	1.78 (0.65 to 4.98)	0.259		
Endocrine, nutritional, metabolic diseases (ICD-10 chapter IV), n (%)	90 (19.2)	22 (19.8)	1.05 (0.62 to 1.80)	0.857		
Diseases of the nervous system (ICD-10 chapter VI), n (%)	75 (16.0)	21 (18.9)	1.31 (0.75 to 2.28)	0.342		
Diseases of the eye/ear (ICD-10 chapters VII/VIII), n (%)	107 (22.9)	22 (19.8)	0.79 (0.47 to 1.34)	0.383		
Diseases of the circulatory system (ICD-10 chapter IX), n (%)	148 (31.6)	42 (37.8)	1.44 (0.92 to 2.25)	0.108		
Diseases of the respiratory system (ICD-10 chapter X), n (%)	62 (13.2)	21 (18.9)	**1.80(1.01to3.20)**	**0.046**	1.31 (0.61 to 2.82)	0.483
Diseases of the digestive system (ICD-10 chapter XI), n (%)	110 (23.5)	25 (22.5)	0.93 (0.56 to 1.55)	0.780		
Diseases of the skin and subcutaneous tissue (ICD-10 chapter XII), n (%)	41 (8.8)	11 (9.9)	1.20 (0.58 to 2.48)	0.624		
Diseases of the musculoskeletal system (ICD-10 chapter XIII), n (%)	123 (26.3)	27 (24.3)	0.87 (0.53 to 1.43)	0.592		
Diseases of the genitourinary system (ICD-10 chapter XIV), n (%)	78 (16.7)	22 (19.8)	1.33 (0.77 to 2.30)	0.309		
Multiple physical conditions, n (%)[Table-fn T4_FN6]	262 (56.0)	71 (64.0)	1.54 (0.99 to 2.40)	0.053		

Bold font indicates statistically significant results. P values and OR were based on a continuous variable for age at death and dichotomous variables in all other analyses. Percentage (%) of suicidality relative to the number of participants with documented suicidality in somatic records during their care (n=111) within each subgroup.

* Missing data: information about civil status for 96 patients and information about employment for 124 patients.

† Documentation of experience of distress in medical records.

‡ Other care providers, as noted in somatic medical records.

§ Diagnosis according to ICD-10, chapter V (codes F00-F99), documented in medical records, used to define psychiatric comorbidity.

¶ Two or more diseases according to ICD-10, chapters I-IV, VI, VII/VIII, IX–XIV, used to define multiple physical conditions.

ICD-10, International Statistical Classification of Diseases and Related Health Problems, 10th Revision.

## Discussion

This study examines the occurrence of documentation of suicidality and associated factors in somatic medical records of those who died by suicide who were in contact with specialised somatic healthcare. Our principal finding was that documentation of suicidality occurring at any point in time during the final 24 months of life was present in the somatic medical records of only about a fourth of the persons who had contact for physical illness on specialised somatic healthcare prior to suicide. Elevated suicide risk was noted in only 6% of the total cohort. The independent factors associated with documentation of suicidality in somatic medical records include female sex, experiences of distress, psychiatric comorbidities and care contact with psychiatric care. No associations were observed for somatic diagnostic categories.

### Strengths and weaknesses

Strengths of the present study include the comprehensive medical record review. Prior to the start of data collection, all local healthcare investigators participated in group training on how to uniformly rate data based on a protocol specific to this study and investigator guidelines. The investigators had a high level of support from the research group during data collection, thereby ensuring consistent assessment and rating of patient data. The chosen method provided rich data on the documentation of suicidality in patients with specialised somatic care prior to suicide. The study had some methodological weaknesses as well. There is no information on whether caregivers adhered to Swedish guidelines for structured suicide risk assessments and the suggested tools^24^ or used alternative tools or clinical practices. Variations in suicidality assessment or tools may have affected clinicians' ability to identify and document suicidality and have potentially influenced the documentation rates observed in this study. The data collection process included no systematic testing of interrater reliability (IRR) of data ratings. A barrier to conducting IRR testing was that the medical record reviewers were clinicians working in different regions of the country. According to the Swedish Public Access to Information and Secrecy Act (SFS: 2009:400), data sharing was not permitted with record reviewers based outside a specific region. Regarding sample selection, patients who died due to self-harm of undetermined intent (ICD-10 codes Y10–Y34) were not included, although many of these were likely suicides.^25^ Nor were patients included if they had diffuse symptoms not classified as a physical disease. The findings in this study are therefore limited in generalisability to patients deceased by certain suicide with at least one diagnosed physical condition.

#### Findings

In this study, we used a very broad definition of documentation of suicidality as the outcome variable, which included both passive suicidal ideation, with death wishes and suicidal thoughts, to active suicidal ideation, with plans for suicide and suicide attempts. We also used documentation about suicidality from all health professionals within specialised somatic care, not only from doctors’ assessments. Despite this broad definition, we only found that 24% had documentation of suicidality during the last 24 months of life. A narrower definition would have yielded an even smaller proportion of patients with documentation of suicidality in connection with consultations in specialised somatic healthcare.

Studies of documentation of suicidality in specialised somatic healthcare are sparse. The proportion (24%) in our study is therefore not comparable with others who reported data for other healthcare settings.^26^ One study assessing suicide ideation in patients at admission to somatic inpatient care^16^ showed that about 7% of the patients disclosed suicide ideation but did not die by suicide. This proportion is similar to that we observed for suicide-related thoughts (8.5%) in this cohort of both outpatients and inpatients who died by suicide.

We found that roughly one-third of the patients in the present study had care contact with a doctor in somatic healthcare during their last month of life, but a majority lacked assessment of suicide risk. This may be due to the limited attention by clinicians to mental health issues in their physically ill patients and to prioritisation of suicide risk assessment to patients with known psychiatric contact or diagnoses. Lack of risk assessment might also be related to the work routines in specialised somatic healthcare. The time spent in a conversation between a doctor in somatic specialised healthcare and the patient, for natural reasons, focuses on other issues than mental health, such as symptoms, treatment and prognosis of the physical illness. Clinicians might lack time, knowledge and skill training in suicide risk assessments, which may make them feel uncomfortable and distance themselves from these tasks.^27^ The lack of documentation may also be a result of denial of suicidality. Of the 29 patients in the present study with documentation of suicide risk assessment in somatic specialist care during their last month of life, 11 disclosed suicidal ideation. It is possible that those who denied, at the time of assessment, had not yet developed suicidality or, alternatively, had fluctuating suicidality and were not experiencing suicidality at the time of the consultation. Research has shown that the period from a decision to act to the actual suicide attempt can be as short as 10 min in about every second person who makes a suicide attempt.^28^ Moreover, we could not ascertain whether patients denied suicidal issues despite having such ideation. A meta-analysis showed that approximately 44% of individuals with suicidal ideation tended to deny its occurrence.^29^ A review investigating suicidal ideation up to 1 month before death by suicide found that the denial rate across studies ranged from 18% to 94%, with the rate of denial differing among different healthcare settings.^30^ Patients may deny suicidality to avoid hospitalisation, which would prevent them from acting on their death wishes.^26^ Some patients in the present study may have perceived their symptoms of suicidality as less severe at the time of contact with specialised somatic care and, therefore, chose not to disclose them. This tendency to minimise the severity of suicidality,^26 31^ or a sense of shame or guilt about being suicidal,^32^ may also result in non-disclosure to a clinician. However, to enable patients to open up and disclose potential suicidality, a good alliance, with a non-judgemental and empathetic attitude towards patients, might be a successful way to overcome obstacles and provide a necessary basis for an open dialogue, facilitating disclosure of suicidality.^33^

#### Sex differences

Documentation of suicidality was more common in women. This specific sex difference has in literature been termed the gender paradox, in that women report higher rates of suicidal ideation and non-fatal suicidal behaviour including suicide attempts, whereas men have higher rates of death by suicide.^34^,^36^ The sex differences in documentation of suicidality in the present study may also be a result of gender differences in disclosing distress. Previous studies have likewise shown that men seeking help in professional care do not often disclose suicidal ideation to their care contact.^34 37^

### Clinical factors associated with documentation of suicidality

Patients having a care contact with psychiatric services or having a psychiatric diagnosis in addition to a diagnosis of a physical condition were found to have a higher likelihood of documented suicidality in their medical records from specialised somatic healthcare services. This was not surprising, as studies investigating lifetime suicidal ideation and disclosure have shown that patients with past suicidal behaviour and diagnosis of a psychiatric disorder have a higher probability of disclosing suicidal ideation to healthcare professionals.^36^

### Implications for healthcare providers

It is important to note that about two thirds of the patients had no psychiatric care contact in the 2 years before suicide. Therefore, clinicians in specialised somatic healthcare services need to be prepared to recognise and address mental health issues or distress in their patients. Reasons for psychological suffering may include feelings of hopelessness, entrapment, defeat and humiliation, as well as concerns about burdening other people,^38 39^ functional disability, compromised autonomy,^1 40^ problems with relationships and loneliness or problems with employment, economy and a lost sense of coherence.^41^ A clinical routine for assessing and documenting patients’ mental health within specialised somatic healthcare services may increase clinicians’ awareness of psychological suffering and could probably result in a higher detection of psychiatric comorbidity and passive or active suicidal ideation. Identifying and documenting suicidality in somatic medical records gives an opportunity to act and offer evidence-based treatment for suicide prevention.^24^ Joint discussions may be needed between different care settings (eg, psychiatric services, different branches of specialised somatic care, primary care and social services) to find ways to collaborate and share responsibilities and to create an effective person-oriented suicide preventive treatment. However, to enable closer collaboration between different care settings, it also requires implications of research findings in a public health context. Such implications might require changes at an organisational level and a recognition that suicide prevention requires cooperation between the entire healthcare system and society. In a recent review aiming to translate suicide research into action, O’Connor and co-authors^42^ advocate a change of organisational boundaries between mental healthcare and somatic healthcare in order to support staff to work on suicide prevention throughout the healthcare system.

## Conclusions and implications for further research

Our findings highlight the need for specialist somatic healthcare staff to target both physical and mental health in their patients. Given the low occurrence of recognition of suicidality in the somatic medical records, future research should investigate barriers to the lack of assessment of mental health. Research should also evaluate whether routine assessment of mental status, including suicidality and experiences of distress in patients on somatic wards and in somatic outpatient clinics, could improve the detection of suicidality and prevent suicidal behaviour or death by suicide in patients with compromised physical health.

## Data Availability

Data are available upon reasonable request.
