# Supplementary material for: Documentation of suicidality in connection with specialised healthcare for physical conditions: a retrospective review of somatic medical records prior to suicide in Sweden
Source: BMJ Open. 2025 May 15;15(5):e086633. doi: 10.1136/bmjopen-2024-086633 (PMC12083290; doi:10.1136/bmjopen-2024-086633)
Supplement: Supplementary file 1 [file bmjopen-2024-086633.reviewer_comments.pdf]

PEER REVIEW HISTORY

BMJ Open publishes all reviews undertaken for accepted manuscripts. Reviewers are asked to complete a checklist review form and are provided with free text boxes to elaborate on their assessment. These free text comments are reproduced below.

ARTICLE DETAILS

Title (Provisional)

Documentation of suicidality in connection with specialised health care for physical conditions: A retrospective review of somatic medical records prior to suicide in Sweden

Authors

Sellin, Tabita; Waern, Margda; Bergqvist, Erik; Palmqvist Öberg, Nina; Probert-Lindström, Sara Maria; Fröding, Elin; Ehnvall, Anna; Westrin, Åsa

---

VERSION 1 - REVIEW

**Reviewer**	**1**
**Name**	**Michaud, Laurent**
**Affiliation**	**Lausanne University Hospital**
**Date**	**09-May-2024**
**COI**	**None**

This paper presents a study aiming to evaluate whether suicidality was documented prior to suicide in patients consulting health care providers for physical conditions, and how several variables could be associated to this documentation. It is an interesting topic, the study is well thought out and I do not have major concern about methods and results. However, I think that the Introduction and Discussion sections should be thoroughly revised: authors should seek to (i) better identify and explicit the gap of knowledge in the introduction (see below) and (ii) discussing more in depth the clinical relevance of their results and links to the literature topic in the discussion. Indeed, readers should be informed on if and how there is a link between the documentation of suicidality and suicide prevention. Actually, documentation is often more a medico-legal and institutional requirement than a clinical and therapeutical concern and it's not sure that it represents a valid proxy of quality of care of suicidal patients. Moreover, as underlined in one of the cited references (Hawton K, Lascelles K, Pitman A, Gilbert S, Silverman M. Assessment of suicide risk in mental health practice: shifting from prediction to therapeutic assessment, formulation, and risk management. Lancet Psychiatry. 2022;9:922-8), focus in suicide prevention should be more

on building a trustful relationship, joint planning ways to cope with crisis, etc… than on “measuring” or “stratify” the suicide risk.

As for the language, I am not a native English speaker, but I feel that having the manuscript edited by a native English speaker could improve its readiness.

Here are my specific comments:

• Introduction focus on the existing literature on links between suicidality and physical conditions. I think this could be far shortened; indeed, the study does not fill a gap in this field. Instead, the introduction could focus on the research concerning suicide documentation and studies on patients deceased by suicide (see for example in a veteran’s sample Ribeiro JD, Gutierrez PM, Joiner TE, Kessler RC, Petukhova MV, Sampson NA, Stein MB, Ursano RJ, Nock MK. Health care contact and suicide risk documentation prior to suicide death: Results from the Army Study to Assess Risk and Resilience in Servicemembers (Army STARRS). J Consult Clin Psychol. 2017 Apr;85(4):403-408. doi: 10.1037/ccp0000178. PMID: 28333538; PMCID: PMC5364817 and cited references, or in a psychiatric sample records our study Stauffacher MWD, Stiefel F, Dorogi Y, Michaud L. Observational study of suicide in Switzerland: comparison between psychiatric in- and outpatients. Swiss Med Wkly. 2022 Feb 28;152:w30140. doi: 10.4414/smw.2022.w30140. PMID: 35230043 and the cited references), and if and how this documentation is relevant for good clinical practice.

• Study design: “Of the 948 individuals in the catchment area who died by certain suicide January 1 to December 31 2015, 630 (66.5%) had been in contact with […] certain suicides in the catchment area were included in the present study (331 men and 137 women). “ This could be in the Results section.

**Reviewer**	**2**
**Name**	**Sperling, Edie**
**Affiliation**	**Western University of Health Sciences, Medical Anatomical Sciences**
**Date**	**10-Sep-2024**
**COI**	**None.**

Thank you for the opportunity to review this very interesting manuscript. It is well-written, covers an important topic, and is methodologically sound. I have a few suggestions for making it more clear to readers who may not be familiar with your previous work, or are from different countries which may have slightly different terminology.

The main edit I have is to define "specialized somatic health care providers" early in the manuscript, including in the Abstract. In my experience, this term could mean almost anything, and it's not defined until page 9. Somatic health care providers in the U.S. tend to be thought of separately from regular specialist physicians, so defining this early is very important. It could be as simple as "specialized somatic health care providers (for example, XXX) etc etc." You may also consider changing "somatic" to "physical" to avoid this problem altogether (but still examples are needed).

Abstract:

-In Abstract Results, please clarify that 111 (24%) were positive for the outcome of Documentation of suicidality whether they were suicidal or not. This was confusing at the time, though you explained it later on.

Strengths and Limitations

-If there is room, please state why you did not/could not do an IRR. I assume it's because there were too many people contributing to the data that it would have been logistically impossible, but this should be stated.

Introduction:

- In the sentence "About 7% of patients with various physical illnesses..." I believe is citing just a single study? If so, please clarify how many patients were assessed altogether and there that study took place.

-Please state why one region was not able to be included in the study (20 of 21 regions).

Results:

-I believe this is the first time you define what a specialized somatic care provider is.

- Pg 9 "These patients differed in one sociodemographic..." please clarify what "these patients" means. ("Patients that saw specialized care providers vs ones that did not")

-P. 12 You may want to clarify that "emotional or trauma-related" is emotional trauma. It is generally clear given the context of that section but could be made perfectly clear.

Findings:

- Could differences in screening questions also have affected a provider's ability to discern the presence of suicidality? Or does everyone use the same screening tool?

VERSION 1 - AUTHOR RESPONSE

**Comments Reviewer 1** Dr. Laurent Michaud	**Response and changes**
Introduction focus on the existing literature on links between suicidality and physical conditions. I think this could be far shortened; indeed, the study does not fill a gap in this field.<br><br>Instead, the introduction could focus on the research concerning suicide documentation and studies on patients deceased by suicide<br><br>(see for example in a veteran's sample Ribeiro JD, Gutierrez PM, Joiner TE, Kessler RC, Petukhova MV, Sampson NA, Stein MB, Ursano RJ, Nock MK. Health care contact and suicide risk documentation prior to suicide death: Results from the Army Study to Assess Risk and Resilience in Servicemembers (Army STARRS). J Consult Clin Psychol. 2017 Apr;85(4):403-408. doi: 10.1037/ccp0000178. PMID: 28333538; PMCID: PMC5364817 and cited references, or in a psychiatric sample records our study Stauffacher MWD, Stiefel F, Dorogi Y, Michaud L. Observational study of suicide in Switzerland: comparison between psychiatric in- and outpatients. Swiss Med Wkly. 2022 Feb 28;152:w30140. doi: 10.4414/smw.2022.w30140. PMID: 35230043 and the cited references),<br><br>…and if and how this documentation is relevant for good clinical practice.	The introduction is now abbreviated.<br><br>New text has been added referring to your suggested references from psychiatric samples (Stauffacher et al 2022) and (Riberio et al 2017) (Page 3, Line 18-23). However, since our study aims to decrease the knowledge gap of documentation in *physically ill* patients before suicide, we added a sentence about care-contacts before suicide in somatic health care in comparison to psychiatric care (page 3, line 23-25(Bergqvist, E. et al, 2022). We have reframed the introduction to better clarify the knowledge gap of documentation of suicidality among physically ill patients.<br><br>In clinical implication section (page 18, line 8-11), we discuss the importance of documentation of suicidality in clinical practice, as a step towards preventive interventions.
**Study design**: "Of the 948 individuals in the catchment area who died by certain suicide January 1 to December 31, 2015, 630 (66.5%) had been in contact with […] certain suicides in the catchment area were included in the present study (331 men and 137 women) ". This could be in the Results section.	The suggestion is in line with the Strobe guideline. This text is now moved from the study design section and added below a new sub-heading: "Participants" in the results section (Page 8, Line 43-page 9, line 6).
**Reviewer 2:** Dr. Edie Sperling	**Responses and changes**
The main edit I have is to define "specialized somatic health care providers" early in the manuscript, including in the Abstract	This is now explained in abstract (page 1, line 12-15) and defined early in the manuscript, in Study design and setting (page 5, line 8-15).
In Abstract Results, please clarify that 111 (24%) were positive for the outcome of *Documentation of suicidality* whether they were suicidal or not. This was confusing at the time, though you explained it later.	This is now clarified in the first sentence of results in Abstract (page 1, line 34).

Strengths and Limitations<br>If there is room, please state why you did not/could not do an IRR. I assume it's because there were too many people contributing to the data that it would have been logistically impossible, but this should be stated.	A sentence is added in strength and limitations to explain why no IRR testing was possible (page 14, line 43-54).
Introduction:<br>- In the sentence "About 7% of patients with various physical illnesses..." I believe is citing just a single study? If so, please clarify how many patients were assessed altogether and there that study took place.<br><br>-Please state why one region was not able to be included in the study (20 of 21 regions).	This is now clarified by adding requested information in introduction (page 3, line 32-39)<br><br>This is now clarified in page 4, line 32-34
Results:<br>-I believe this is the first time you define what a specialized somatic care provider is.<br><br>Pg 9 "These patients differed in one sociodemographic..." please clarify what "these patients" means. ("Patients that saw specialized care providers vs ones that did not")<br><br>Pg 12 You may want to clarify that "emotional or trauma-related" is emotional trauma. It is generally clear given the context of that section but could be made perfectly clear.	Specialised somatic care is now exemplified in the Abstract (page 1, line 12-15) and defined early in the manuscript, in Study design and setting (page 5, line 8-15).<br><br>This is a comparison between those with emergency care visits only and those in another specialised somatic clinic. This is clarified in page 10,line 18-25.<br><br>It is not just emotional trauma that is meant here. This is now clarified: distress due to trauma (both emotional and physical) in methods, page 6, line 52-55) and in results page 12 (line 27),
Findings:<br>- Could differences in screening questions also have affected a provider's ability to discern the presence of suicidality? Or does everyone use the same screening tool?	This is mentioned in the “Strength and limitation section” (page 14, line 33-43).
Both reviewers: If you have selected Yes above, pleas add a statement of Competing interest: None	A statement of Competing interest ‘None’ is added in submission process. This is also stated in the manuscript (page 2, line 41-43).

VERSION 2 - REVIEW

Reviewer 2

Name Sperling, Edie

Affiliation Western University of Health Sciences, Medical Anatomical Sciences

Date 28-Jan-2025

COI

Thank you for the opportunity to review this manuscript. All of my questions and suggestions have been addressed by the authors and I now find the manuscript acceptable for publication.
